# Exercise interventions and multidimensional cognitive function in esports players: a systematic review and meta-analysis of randomized controlled trials

**DOI:** 10.3389/fpsyg.2026.1910084

**Published:** 2026-08-14

**Authors:** Haowen Sun, Jiayi Yao, Yongliang Zhu

**Affiliations:** 1 Department of Mathematics, City University of Hong Kong, Hong Kong, Hong Kong SAR, China; 2 School of Physical Education, China University of Mining and Technology, Xuzhou, China; 3 School of Physical Education, Shandong Sport University, Jinan, China

**Keywords:** cognitive function, esports players, exercise intervention, meta-analysis, randomized controlled trials, systematic review

## Abstract

**Background and objective:**

Esports places stringent demands on participants in terms of neural information processing speed, real-time decision-making precision, and cognitive stability. The coexistence of prolonged sedentary training patterns and sustained high-intensity mental workload has rendered the maintenance of cognitive function in esports populations an urgent scientific concern. This study employs systematic review and meta-analysis methods to quantitatively synthesize the effects of exercise interventions on multidimensional cognitive function in esports players, with the aim of providing evidence-based references for the scientific training of esports athletes.

**Methods:**

A systematic search was conducted across PubMed, Embase, Web of Science, Cochrane Library, and Scopus from database inception to May 3, 2026, to identify randomized controlled trials (RCTs) that investigated physical activity interventions among esports players. Two independent researchers completed literature screening and data extraction. The RoB 2.0 tool was used to assess risk of bias, and standardized mean differences (SMDs) with 95% confidence intervals (CIs) were synthesized using a random-effects model.

**Results:**

A total of 5,298 records were identified through database searching, and 10 randomized controlled trials involving 503 participants were ultimately included. Regarding cognitive speed, exercise intervention significantly reduced players’ reaction time (SMD = 0.84, 95% CI: 0.50–1.18), with subgroup analysis indicating that VR/exergame interventions yielded a numerically higher effect size (SMD = 1.01) compared to traditional aerobic exercise (SMD = 0.64). Regarding operational accuracy, the intervention effect was robust and highly consistent across conditions (SMD = 0.58, 95% CI: 0.40–0.76, I^2^ = 0%), remaining stable across both intervention media (traditional 0.53 vs. VR 0.64) and intervention duration (acute 0.57 vs. long-term 0.59). Regarding executive function and memory, the pooled effect size after excluding high-risk studies was SMD = 0.63 (95% CI: 0.36–0.91, I^2^ = 0%), suggesting a robust promoting effect of exercise intervention on higher-order cognitive function. Sensitivity analyses confirmed that core outcome effects remained significant after removing sources of heterogeneity, and Egger’s test indicated no significant publication bias for the accuracy outcome (*p* = 0.54).

**Conclusion:**

Current evidence suggests that exercise intervention is a potentially effective approach for improving multidimensional cognitive function in esports players, with positive effects observed across cognitive processing speed, operational precision, and executive function and memory. VR/exergame interventions showed a numerically larger effect size for cognitive processing speed compared with traditional exercise modalities; however, this difference should be interpreted cautiously due to the exploratory nature of subgroup analyses. Future esports training systems may consider incorporating structured exercise interventions into routine training programs, with the aim of maintaining players’ cognitive health while exploring feasible pathways for extending career longevity.

**Systematic review registration:**

www.crd.york.ac.uk/prospero, identifier [CRD420261385557].

## 1 Introduction

The professionalization of esports has been advancing at an accelerating pace, and this competitive format places stringent demands on participants in terms of neural information processing speed, real-time decision-making capacity, and psychological resilience under pressure (Smith et al., 2022). The in-game performance of professional players has therefore become a key dimension for evaluating individual competitive ability (Sharpe et al., 2023), while the vast population of semi-professional and high-level amateur players is equally subject to the health pressures accompanying this trend toward professionalization. From a cognitive science perspective, the specialized competencies of esports players are not determined solely by operational proficiency; higher-order executive functions including inhibitory control, working memory, and cognitive flexibility are equally central to competitive performance (Valls-Serrano et al., 2022). Recent evidence further suggests that esports participation itself may induce specific cognitive and neurophysiological adaptations. Structured esports training has been associated with improvements in planning and decision-making abilities, while neurophysiological investigations have identified alterations in brain activity patterns related to cognitive control, attention, and decision processes among individuals engaged in esports activities (Imanian et al., 2025a; Imanian et al., 2025b). Comparative studies have also indicated that esports participants may demonstrate cognitive performance characteristics comparable to traditional sports participants, although they do not obtain equivalent benefits in physical fitness and postural control (Leonte et al., 2026). However, prolonged sedentary training in fixed postures, the sustained accumulation of mental fatigue, and a systematic absence of physical activity collectively constitute occupational health risks prevalent within this population (DiFrancisco-Donoghue et al., 2019), and are considered important potential contributors to cognitive decline, reduced decision-making efficiency, and shortened career longevity (Poulus et al., 2020; Giakoni-Ramírez et al., 2022). Although conventional understanding has regarded extensive specialized practice as the primary pathway to competitive advancement (Pluss et al., 2021), emerging evidence indicates that deterioration in basic physiological function may indirectly constrain players’ neural responsiveness (Lam et al., 2022), making the exploration of adjunctive interventions to maintain overall competitive capacity an industry-level practical necessity (Emara et al., 2020).

Among existing intervention strategies, exogenous nutritional supplementation remains the most prevalent regulatory approach in esports, with caffeine, taurine, and various nootropic supplements widely used to counteract the cognitive depletion associated with high-intensity competition (Yao et al., 2026; Szot et al., 2022). Such approaches have gained broad acceptance among players owing to their ease of use. Nevertheless, long-term reliance on supplementation alone carries notable limitations: sustained adherence is difficult to maintain, potential adverse effects cannot be overlooked, and the capacity to comprehensively improve physiological function through a single intervention modality remains restricted (Seesurn et al., 2025; Sainz et al., 2020). By contrast, physical activity, as an endogenous active regulatory strategy, demonstrates distinctive advantages in promoting neuroplasticity and enhancing cognitive performance (Gram et al., 2014). At the mechanistic level, exercise stimulation may upregulate the secretion of brain-derived neurotrophic factor (BDNF) and modulate cortical arousal, thereby providing physiological support for the sustained and stable performance of esports players under conditions of high cognitive load (da Silva et al., 2017). Exercise interventions in esports populations can generally be classified into conventional exercise modalities and emerging VR/exergame-based approaches. Conventional exercise interventions primarily include aerobic training, resistance training, and high-intensity interval training, whereas VR/exergame interventions combine physical activity with interactive digital environments. Given the technology-oriented characteristics of esports, VR/exergame approaches have received increasing attention as a potentially suitable exercise modality for this population; however, whether they provide additional cognitive benefits compared with conventional exercise remains to be further investigated.

Although research examining the relationship between physical activity and esports performance is gradually accumulating, the existing evidence base exhibits notable deficiencies across multiple dimensions. First, prior reviews have been predominantly descriptive in nature, lacking systematic quantification of effect sizes, and meta-analyses specifically targeting professional, semi-professional, and high-level amateur esports populations remain absent (McNulty et al., 2023). Second, emerging intervention modalities such as VR exercise and motion-sensing interactive training have progressively entered the esports training landscape, yet whether they confer differential advantages over traditional aerobic exercise in improving cognitive processing speed remains to be examined through systematic multi-study integration (Tholl et al., 2026). Third, existing primary studies are generally characterized by limited sample sizes and high heterogeneity in intervention protocols, leaving the development of standardized exercise prescriptions insufficiently supported by available data (Huang et al., 2026). Although recent randomized controlled trial protocols have begun to investigate the effects of structured exercise programs on physical activity and cognitive performance among esports athletes, empirical evidence from completed intervention studies remains limited (Tomás et al., 2016; Thillier et al., 2023), the underlying logic by which physical activity acts on cognitive function through the optimization of neural processing efficiency currently relies largely on inferences drawn from dispersed behavioral data, and its robustness awaits verification through systematic review and sensitivity analysis.

In response to these research gaps, the present study was designed to address the following research question: whether exercise interventions improve multidimensional cognitive function in esports players compared with non-exercise controls. We hypothesized that exercise interventions would produce beneficial effects across multiple cognitive domains, including cognitive speed, operational accuracy, and executive function and memory. The present study employs systematic review and meta-analysis methods to comprehensively evaluate the aggregate effects of exercise interventions on multidimensional cognitive function in esports players. The study focuses on quantifying effect sizes across three dimensions: cognitive speed, operational accuracy, and executive function and memory, while systematically comparing the differential benefits associated with different intervention media and durations. Through the integrated analysis of existing randomized controlled trial data, this study aims to provide empirical references for the optimization of cognitive function among competitive esports players with different levels of experience, and to offer evidence-based support for the future development of scientifically grounded and individualized exercise programs targeting esports players.

## 2 Materials and methods

The protocol for this study was prospectively registered in PROSPERO (registration number: CRD420261385557), specifying the research objectives, inclusion and exclusion criteria, intervention and comparator conditions, and outcome indicators. The study was conducted in strict accordance with the pre-registered protocol without major deviations, and was implemented and reported following the Preferred Reporting Items for Systematic Reviews and Meta-Analyses (PRISMA 2020) checklist (Page et al., 2021).

### 2.1 Search strategy

In accordance with PRISMA 2020 guidelines, a systematic literature search was conducted across five databases: PubMed, Embase, Web of Science, Cochrane Library, and Scopus, from database inception to May 3, 2026. Only studies published in English were eligible for inclusion. The search framework was constructed using a combination of controlled vocabulary and free-text terms, organized around three core dimensions. The population dimension incorporated terms related to esports athletes, competitive-level players, and habitual long-term gaming participants. The intervention dimension focused on various forms of exercise intervention, encompassing aerobic training, resistance exercise, high-intensity interval training, and virtual reality interactive training incorporating physical activity components. The study design dimension was restricted to randomized controlled trials. Terms within each dimension were combined using the Boolean operator “OR,” while dimensions were intersected using “AND.” The complete step-by-step search strategies for each database are provided in Supplementary material text 1.

### 2.2 Inclusion and exclusion criteria

Inclusion and exclusion criteria were established in accordance with the PICOS framework. Inclusion criteria required that participants be experienced esports players, including professional, semi-professional, or high-level amateur players with established gaming experience; that interventions comprise aerobic exercise, resistance training, high-intensity interval training, or exergames incorporating physical activity components; that comparators consist of passive rest, habitual lifestyle, or sedentary tasks; that outcome measures include standardized assessments of cognitive speed, accuracy, or executive function and memory; and that the study design was a randomized controlled trial, as RCTs provide the strongest evidence for evaluating the causal effects of exercise interventions while minimizing potential confounding and selection bias. Participants without established gaming experience (i.e., novice gamers) were excluded because cognitive changes observed in these populations may primarily reflect game familiarization or learning effects rather than adaptations associated with competitive esports participation. Exclusion criteria encompassed non-randomized controlled designs, participants who were non-players or clinical samples with neurodevelopmental disorders, interventions lacking active physical activity components, and studies from which data could not be extracted due to missing data or limitations in data presentation.

### 2.3 Data collection

Data extraction was conducted independently by two researchers using a pre-established standardized extraction form, with any discrepancies resolved through discussion between the two reviewers or by consulting a third senior researcher. The core extracted information included study characteristics such as first author, publication year, sample size, participant demographic characteristics, and competitive level, as well as specific parameters of the physical activity intervention including exercise type, intensity, frequency, and duration. For outcome measures, the means and standard deviations of both the experimental and control groups at baseline and post-intervention were systematically recorded. For studies with incomplete data, attempts were made to contact the original authors; where data could not be obtained, conversion or estimation was performed using methods recommended in the Cochrane Handbook (Wan et al., 2014).

### 2.4 Risk of bias and certainty of evidence

The risk of bias in included studies was independently assessed by two researchers using the Cochrane Collaboration’s Risk of Bias tool for randomized trials (RoB 2.0; Sterne et al., 2019). Assessment covered five domains: the randomization process, deviations from intended interventions, missing outcome data, measurement of outcomes, and selective reporting of results. Each domain was rated as low risk, some concerns, or high risk. Discrepancies between the two assessors were resolved through group discussion or adjudication by a third senior researcher.

### 2.5 Certainty of evidence

The certainty of evidence for core outcome indicators was assessed using the GRADE system (Guyatt et al., 2011). This system considers the risk of bias in included studies, inconsistency attributable to heterogeneity, indirectness of evidence, imprecision of results, and potential publication bias. Based on these criteria, the overall quality of evidence was classified into four levels: high, moderate, low, and very low.

### 2.6 Data analysis

All statistical analyses were performed using Review Manager 5.4. Given the inherent clinical heterogeneity among included studies in terms of intervention format, participant characteristics, and measurement instruments, a random-effects model was uniformly applied for quantitative synthesis to ensure the robustness of results. Effect sizes were expressed as standardized mean differences (SMDs) with 95% confidence intervals (CIs). For directionally negative indicators such as reaction time, values were reversed prior to analysis by multiplying means by −1, so that all positive SMD values consistently indicated a promoting effect of physical activity on cognitive function. Heterogeneity was quantitatively evaluated using a combination of the Q test and the I^2^ statistic. For robustness verification, sensitivity analyses were conducted using a leave-one-out approach. Exploratory subgroup analyses were further implemented for variables including intervention duration and intervention medium, to identify potential sources of heterogeneity and compare effect differences across conditions.

## 3 Results

### 3.1 Study selection

**Figure 1:**
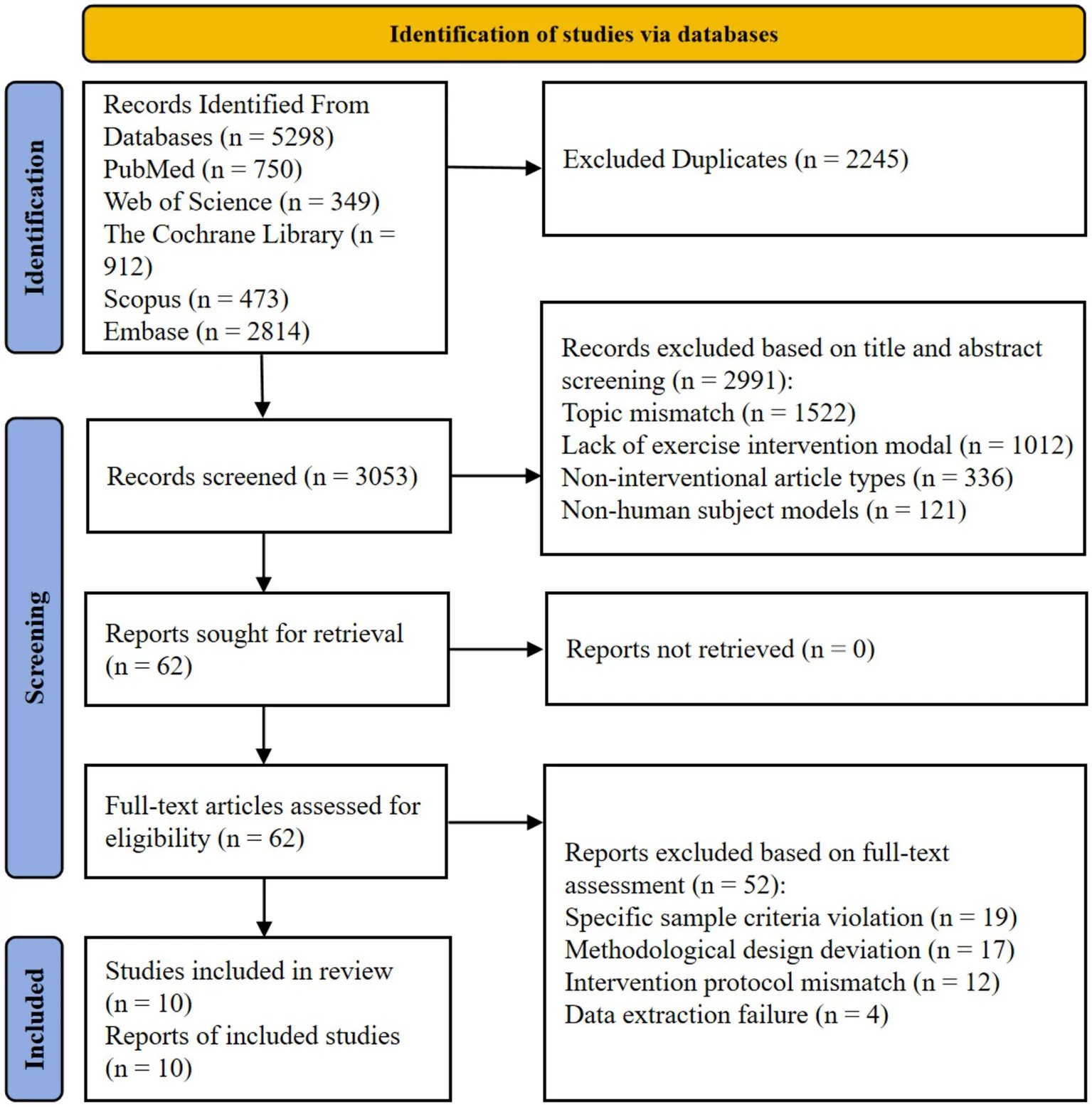
Preferred reporting items for systematic reviews and meta-analysis (PRISMA) study flow diagram.

Literature screening was conducted in strict accordance with PRISMA 2020 guidelines. A systematic database search retrieved an initial pool of 5,298 records, with the following distribution across databases: Embase (*n* = 2,814), Cochrane Library (*n* = 912), PubMed (*n* = 750), Scopus (*n* = 473), and Web of Science (*n* = 349). Automated de-duplication was first performed using EndNote reference management software, followed by manual record-by-record verification by two researchers, resulting in the removal of 2,245 duplicate records and the retention of 3,053 unique citations. Two researchers then independently screened titles and abstracts, excluding 2,991 records for the following reasons: irrelevant research topic (*n* = 1,522), absence of an exercise intervention modality (*n* = 1,012), non-interventional publication type (*n* = 336), and non-human subject models (*n* = 121). Full-text retrieval was subsequently attempted for the remaining 62 records, all of which were successfully obtained. These records then underwent in-depth full-text eligibility assessment, resulting in the exclusion of 52 studies for the following reasons: failure to meet population-specific criteria (*n* = 19), methodological design deviations (*n* = 17), intervention protocol mismatch (*n* = 12), and failure of data extraction (*n* = 4). Ultimately, 10 eligible independent randomized controlled trials (RCTs, comprising 10 reports in total) were included in the systematic review and meta-analysis (Figure 1).

### 3.2 Study characteristics

This meta-analysis included 10 RCTs (He et al., 2025; Lachowicz et al., 2024a; De las Heras et al., 2020; Lachowicz et al., 2024b; Lachowicz et al., 2025a; Zhang et al., 2023; Tang et al., 2025; Lachowicz et al., 2025b; Dos Santos et al., 2024; Mancı et al., 2024) involving a total of 503 participants. Sample sizes across individual studies ranged from 19 to 106, and publication years were concentrated between 2020 and 2025. Participants were predominantly young esports players aged 18 to 28 years, spanning a wide range of competitive levels including top-tier professional players, semi-professional players, and high-level amateur players. Intervention protocols exhibited notable variability across multiple dimensions. With respect to intervention duration, 4 studies employed single-session acute exercise protocols lasting 15 to 30 min, while the remaining 6 studies implemented long-term systematic training programs lasting 4 to 8 weeks. With respect to intervention medium, 4 studies utilized high-immersion VR or motion-sensing interactive exercise, while the remaining studies adopted traditional intervention modalities including moderate-intensity aerobic exercise, HIIT, or resistance training. Outcome measures encompassed three dimensions: cognitive speed, operational accuracy, and executive function and memory. Detailed characteristics of each included study are presented in Table 1.

**Table 1:** Summary of included study characteristics.

Study ID	Participant characteristics (Level, N, nE/nC, Sex)	Age (years) (M ± SD)	Intervention protocol (FITT description)	Control condition	Primary outcome measures	Assessment tools/platform
Tang et al. (2025)	Amateur FPS players, *N* = 83 (nE = 41, nC = 42), 83% male	E:22.15 ± 2.74 C:21.21 ± 2.51	3 sessions/week, 6 weeks; 4 × 4 min HIIT per session	Maintain habitual daily lifestyle	Tracking accuracy, reaction speed	Aim Lab, cycle ergometer
He et al. (2025)	Esports players, *N* = 128 (nE = 32, nC = 32), 100% male	18.0–26.0 (range)	Once daily, 2 weeks; whole-body vibration platform training	Maintain habitual physical activity level	Cognitive processing speed (DSST)	DSST scale
De las Heras et al. (2020)	Video game players, *N* = 20 (nE = 20, nC = 20), 90% male	22.0 ± 3.0 (total sample)	Single session, 15–20 min HIIT	Rest control	Executive control ability	iPad cognitive assessment battery
Zhang et al. (2023)	Esports players, *N* = 34 (nE = 34, nC = 34), 82% male	E:18.59 ± 1.00 C:18.24 ± 0.44	Single session, 30 min cycle ergometer exercise (64–76% HRmax)	Rest control	Reaction speed, cognitive accuracy, visual memory	Human Benchmark platform
Mancı et al. (2024)	Amateur esports players, *N* = 19 (nE = 10, nC = 9), 100% male	E:27.40 ± 3.09 C:26.55 ± 5.05	Single session, high-intensity sprint exercise	Age-matched non-player control	Inhibitory control (Go/No-go)	fNIRS monitoring
Dos Santos et al. (2024)	Experienced FPS players, *N* = 20 (nE = 20, nC = 20), 100% male	19.8 ± 2.5 (total sample)	Single session, 20 min cycle ergometer warm-up (65–75% HRmax)	Rest control	Task completion time, shooting accuracy	CS: GO custom map
Lachowicz et al. (2024a), Brain	Amateur esports players, *N* = 66 (nE = 32, nC = 34), 68% male	E:23.96 ± 3.90 C:23.79 ± 2.67	8 consecutive days, 15 min VR rhythm training/day	Passive control (habitual activity)	Reaction time, motor time, eye-hand coordination	VR device, Vienna Test System
Lachowicz et al. (2024b), SciRep	Amateur esports players, *N* = 66 (nE = 32, nC = 34), 68% male	E:23.96 ± 3.90 C:23.79 ± 2.67	8 consecutive days, 15 min VR rhythm training/day	Passive control (habitual activity)	Attention performance, alternating attention	Cognitrone test, CTT trail making
Lachowicz et al. (2025a), Appl	Amateur esports players, *N* = 128 (nE = 62, nC = 66), 63% male	23.92 ± 3.55 (total sample)	14/42 consecutive days, 15 min VR rhythm training/day	Phased passive control	Cognitive function stability, long-term eye-hand coordination gains	Immersive VR device, VTS system
Lachowicz et al. (2025b), VR	Amateur esports players, *N* = 128 (nE = 62, nC = 66), 63% male	23.92 ± 3.55 (total sample)	8/28 consecutive days, 15 min VR rhythm training/day	Phased passive control	Visuospatial working memory span (VSWM)	Corsi block test (Vienna system)

### 3.3 Risk of bias

**Figure 2:**
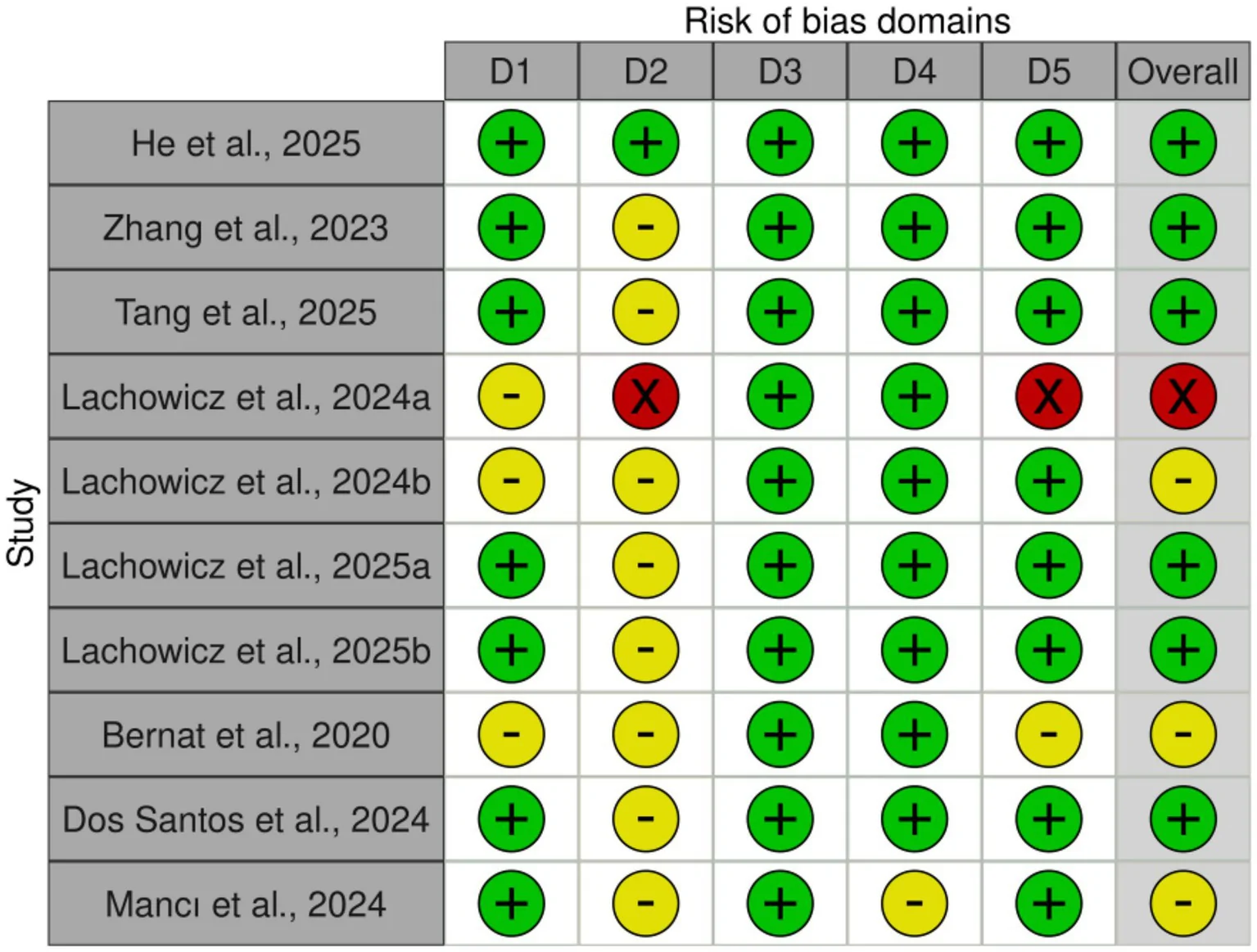
Risk of bias summary: review of the authors’ judgments about each risk of bias item for each included study.

**Figure 3:**
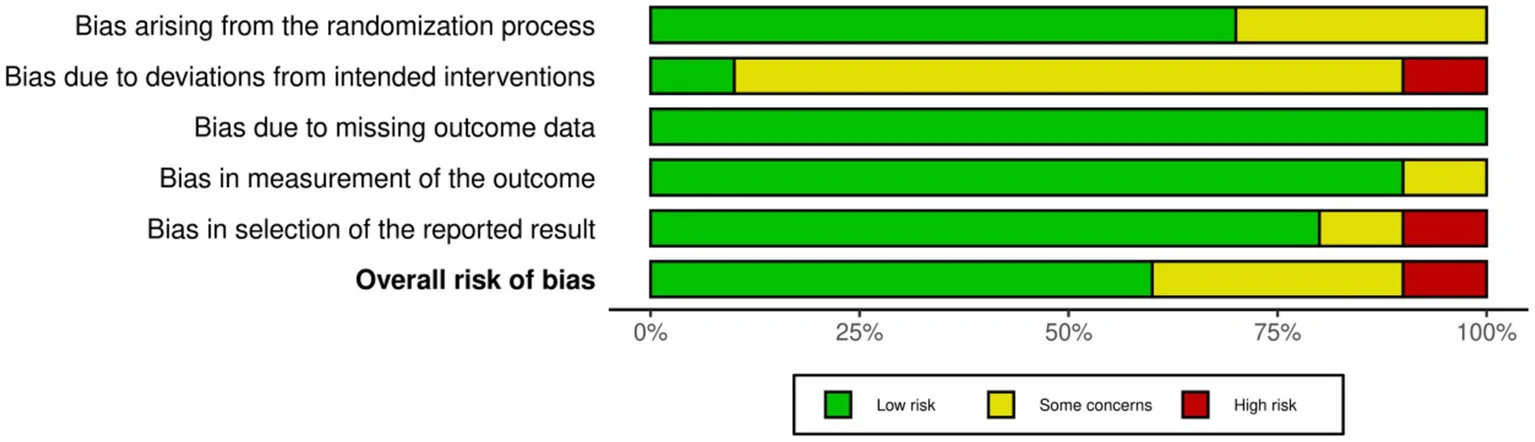
Risk of bias graph: review authors’ judgments about each risk of bias item, presented as percentage of included studies.

The 10 included RCTs exhibited a gradient distribution in risk of bias assessment results. According to the RoB 2.0 framework, three studies, namely He et al. (2025), Zhang et al. (2023), and Tang et al. (2025), were classified as low risk, primarily on the basis of the rigor of their randomization sequence generation, the adequacy of allocation concealment, and the effective implementation of blinded outcome assessment. The remaining six studies were rated as presenting some concerns, with the source of concern concentrated in Domain 2 (deviations from intended interventions), attributable to the methodological difficulty of implementing participant blinding in physical activity interventions. Lachowicz et al. (2024a) was individually rated as high risk, with the primary concern being insufficient control over participants’ expectancy effects, compounded by the absence of prospective protocol registration, which raised legitimate questions regarding selective outcome reporting. Although risk levels varied across studies, the direction of effect sizes remained highly consistent across all included studies. Subsequent sensitivity analyses confirmed that core effects remained significant after excluding the high-risk study, indicating that the gradient distribution of risk of bias did not pose a substantial threat to the robustness of the primary conclusions of this review (Figures 2, 3).

### 3.4 Meta-analysis results

#### 3.4.1 Cognitive speed

**Figure 4:**
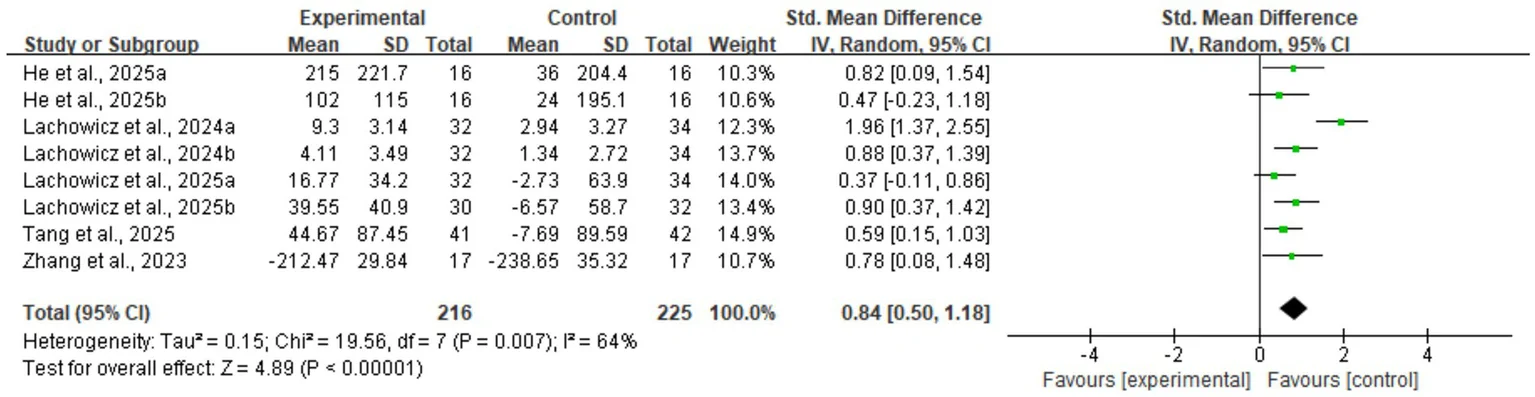
Forest plot of the effects of exercise intervention on cognitive speed in esports players.

Eight independent comparisons involving 541 participants were included, with results presented in Figure 4. The random-effects model indicated that the improvement in cognitive speed was significantly greater in the intervention group than in the control group (SMD = 0.84, 95% CI: 0.50–1.18, *p* < 0.00001). The initial pooled analysis revealed moderate heterogeneity (I^2^ = 64%, *p* = 0.007), and Lachowicz et al. (2024a) was identified as the primary source. Following exclusion of this study, the pooled effect size narrowed to SMD = 0.67 (95% CI: 0.46–0.88, *p* < 0.00001) with heterogeneity completely eliminated (I^2^ = 0%), confirming a robust effect of exercise on reducing reaction time and improving cognitive processing speed.

#### 3.4.2 Accuracy

**Figure 5:**
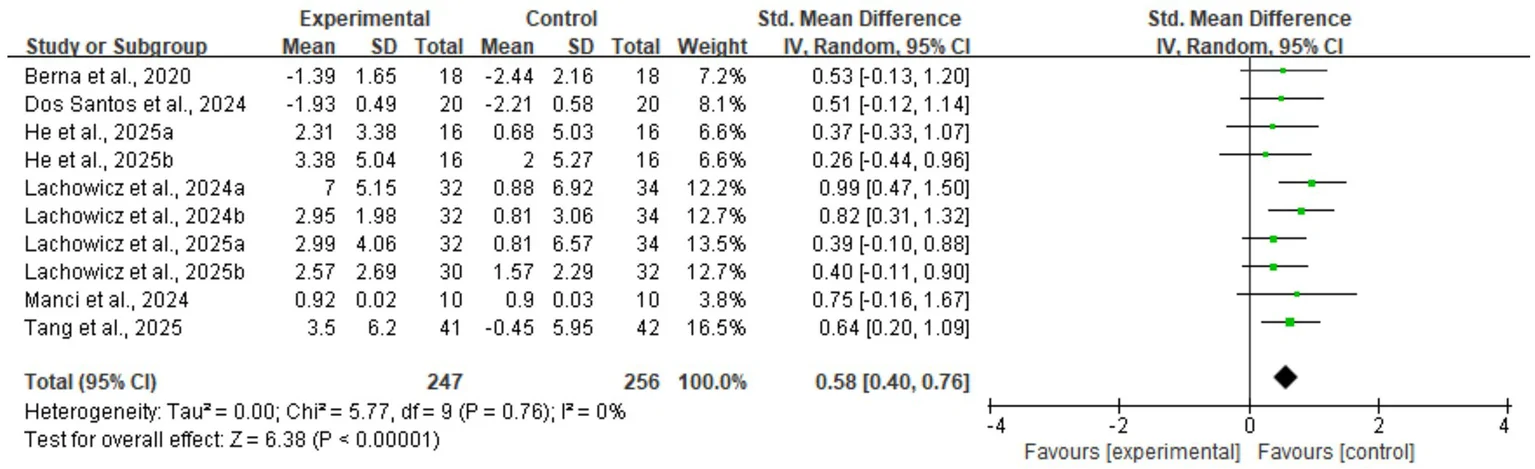
Forest plot of the effects of exercise intervention on accuracy in esports players.

Ten controlled comparisons involving 503 participants were included, with results presented in Figure 5. Statistical heterogeneity across studies was negligible (I^2^ = 0%, *p* = 0.76). The pooled result demonstrated that operational accuracy was significantly improved in the intervention group compared with the control group (SMD = 0.58, 95% CI: 0.40–0.76, *p* < 0.00001), indicating a consistent and stable promoting effect of physical activity on task execution precision in esports players.

#### 3.4.3 Executive function and memory

**Figure 6:**
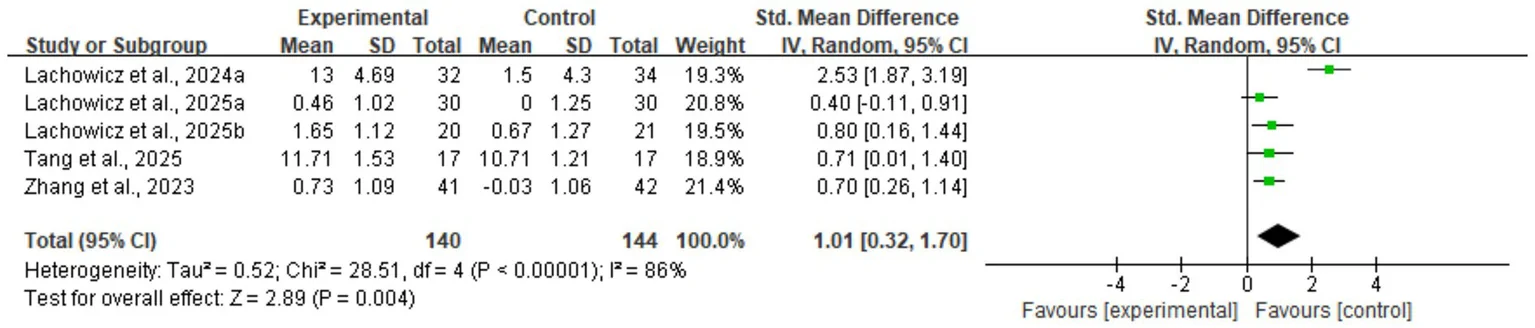
Forest plot of the effects of exercise intervention on executive function and memory in esports players.

Five independent comparisons involving 284 participants were included, with results presented in Figure 6. The pooled effect size indicated that improvements in executive function and memory were significantly greater in the intervention group than in the control group (SMD = 1.01, 95% CI: 0.32–1.70, *p* = 0.004), although substantial heterogeneity was present in the initial analysis (I^2^ = 86%). Following identification and exclusion of Lachowicz et al. (2024a), the pooled effect size was adjusted to SMD = 0.63 (95% CI: 0.36–0.91, *p* < 0.00001) with heterogeneity completely eliminated (I^2^ = 0%), suggesting that the promoting effect of physical activity on higher-order cognitive function is of considerable robustness.

### 3.5 Exploratory subgroup analyses

To further investigate potential sources of heterogeneity and examine the consistency of findings across different intervention conditions, exploratory subgroup analyses were conducted for cognitive speed, accuracy, and executive function and memory. Results are summarized in Table 2. For cognitive speed, the pooled effect size of VR/exergame interventions (SMD = 1.01) was numerically higher than that of traditional exercise modalities (SMD = 0.64); however, this difference should not be interpreted as evidence of superiority because the between-subgroup difference was not statistically significant (*p* = 0.29). For accuracy, subgroup analyses showed consistent beneficial effects across intervention types and durations, with no significant between-group differences observed. In the executive function and memory dimension, the executive function subgroup showed a markedly larger effect estimate than the memory function subgroup (SMD = 2.53 vs. 0.63); however, this finding should be interpreted cautiously because the executive function subgroup included only one study. Detailed forest plots for each subgroup are provided in Supplementary Figures 1–5.

**Table 2:** Summary of exploratory subgroup analysis results.

Outcome	Subgroup dimension	Subgroup category	No. of studies (N)	SMD (95% CI)	P (within group)	I^**2**^ (%)	Between-group difference (P)
Cognitive speed	Intervention medium	Traditional physical activity	4	0.64 [0.34, 0.94]	<0.0001	0%	0.29
		VR/exergame	4	1.01 [0.39, 1.63]	0.001	82%	
Accuracy	Measurement dimension	In-game performance	4	0.60 [0.30, 0.90]	<0.0001	0%	0.87
		Laboratory cognitive test	6	0.57 [0.33, 0.81]	<0.0001	10%	
	Intervention type	Traditional physical activity	6	0.53 [0.27, 0.78]	<0.0001	0%	0.56
		VR/exergame	4	0.64 [0.35, 0.94]	<0.0001	28%	
	Intervention duration	Acute/pre-competition priming	3	0.57 [0.16, 0.98]	0.007	0%	0.93
		Long-term training	7	0.59 [0.39, 0.79]	<0.00001	0%	
Executive function and memory	Cognitive domain	Memory function	4	0.63 [0.36, 0.91]	<0.0001	0%	<0.0001
		Executive function	1	2.53 [1.87, 3.19]	<0.0001	-	

SMD, standardized mean difference; CI, confidence interval. Subgroups containing only one study did not have within-subgroup I^2^ calculated. Detailed forest plots are provided in the supplementary materials.

### 3.6 Publication bias

**Figure 7:**
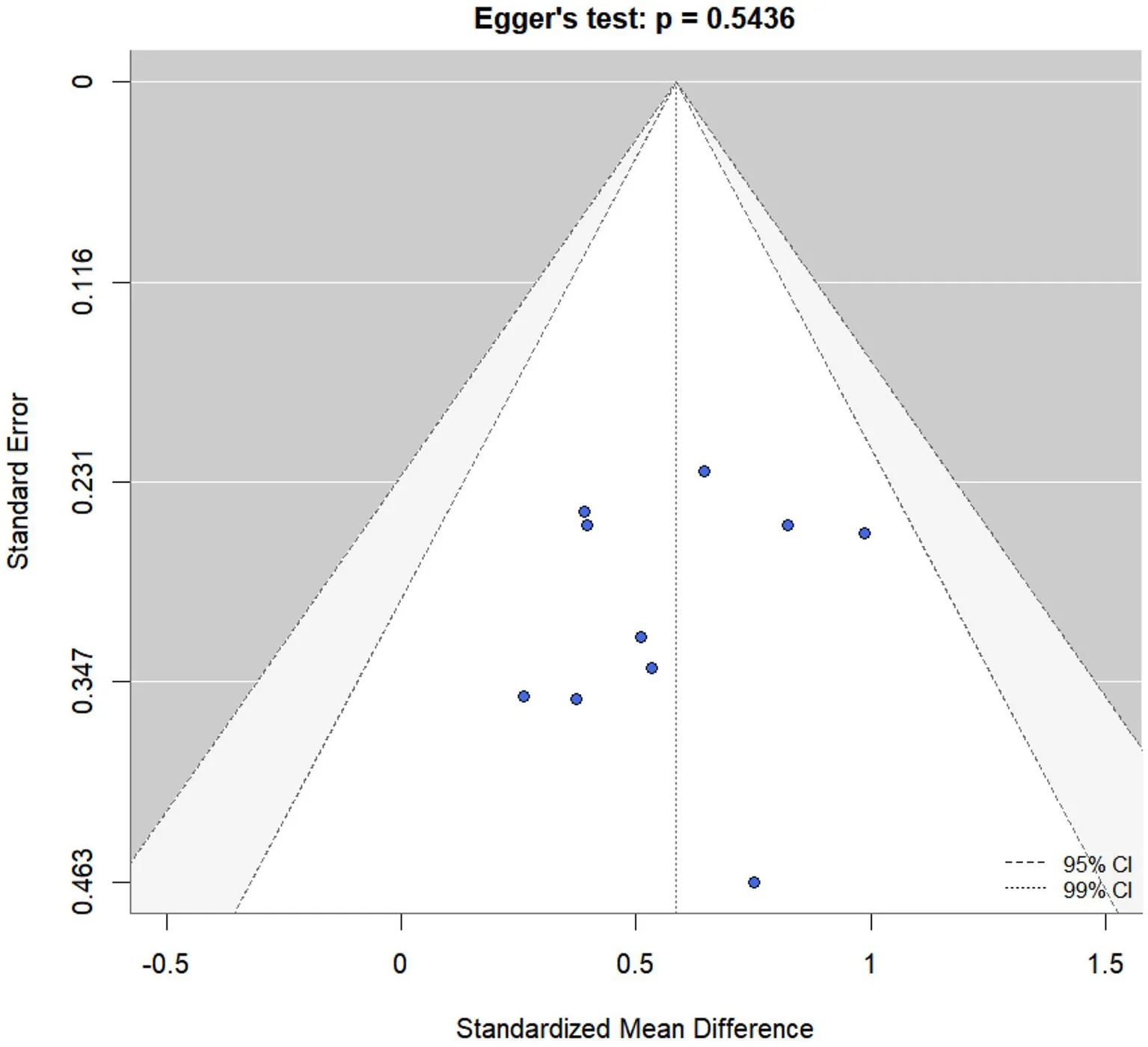
Funnel plot for publication bias assessment and Egger’s test results.

For the accuracy outcome, which comprised 10 included studies, publication bias was assessed using a funnel plot and Egger’s test. The funnel plot for the accuracy outcome displayed an approximately symmetric distribution (Figure 7), and Egger’s test indicated no significant publication bias (*p* = 0.5436). For the remaining outcomes with fewer than 10 included studies, quantitative bias detection was not performed given insufficient statistical power.

### 3.7 Assessment of evidence certainty

GRADE ratings indicated that the certainty of evidence for each outcome in this study was overall at a moderate to low level (Table 3). The accuracy outcome was rated as moderate-certainty evidence on the basis of its robust effect size, high consistency across studies (I^2^ ≤ 10%), and the absence of significant publication bias on Egger’s test. Both cognitive speed and executive function and memory were downgraded to low-certainty evidence due to the inclusion of a high-risk study and the presence of substantial statistical heterogeneity (I^2^ > 60%).

**Table 3:** Evidence certainty ratings.

Outcome	No. of studies	Risk of bias	Inconsistency	Indirectness	Imprecision	Publication bias	Evidence level
Cognitive speed	8	Serious^1^	Serious^2^	None	None	Not assessed^3^	Low
Accuracy	10	Serious^1^	Not significant	None	None	None	Moderate
Executive function and memory	5	Serious^1^	Serious^2^	None	None	Not assessed^3^	Low

1 The inclusion of a high-risk study adversely affected the overall bias rating for these outcomes.

2 Statistical heterogeneity across studies was substantial (I^2^ > 60%), indicating considerable variability in results.

3 For outcomes with fewer than 10 included studies, quantitative publication bias detection was not conducted in accordance with guideline recommendations.

## 4 Discussion

### 4.1 Summary of findings

The present systematic review and meta-analysis aimed to determine whether physical activity interventions improve multidimensional cognitive function in esports players compared with non-exercise controls, with a particular focus on cognitive speed, operational accuracy, and executive function and memory. By synthesizing data from 10 randomized controlled trials, this study provides quantitative evidence regarding the potential cognitive benefits of exercise interventions in esports populations. Overall, physical activity demonstrated statistically significant positive effects across all three cognitive dimensions. The improvement in operational accuracy showed high consistency across studies (SMD = 0.58, I^2^ = 0%), with effect sizes remaining stable regardless of intervention medium (traditional exercise or VR/exergame) and intervention duration (acute or long-term). Regarding cognitive processing speed, physical activity significantly reduced reaction time (SMD = 0.84), with the effect size observed under VR/exergame interventions (SMD = 1.01) being numerically higher than that of traditional aerobic exercise (SMD = 0.64), suggesting that VR-based approaches may represent a promising modality for enhancing cognitive outcomes in esports players. For executive function and memory, the pooled effect size after excluding the high-risk study was SMD = 0.63 (I^2^ = 0%), indicating a robust beneficial effect of physical activity on higher-order cognitive abilities. Although some outcomes were influenced by heterogeneity and risk of bias, resulting in moderate-to-low certainty of evidence, the consistent direction of effects across cognitive domains supports the potential role of physical activity as an effective strategy for improving cognitive function in esports players.

### 4.2 Comparison with existing literature and mechanistic analysis

The findings of the present study are broadly consistent with the overall direction of prior exploratory reviews in this field, while providing a more precise evidence base through effect size quantification and subgroup stratification (McNulty et al., 2023). With respect to cognitive speed, the reaction time reduction effect observed in this study (SMD = 0.84) exceeds the average level reported in studies involving general populations, which may reflect the higher neuroplasticity baseline established through long-term specialized training in esports populations, rendering them more sensitive to exercise-induced changes in cortical arousal (Bediou et al., 2018; Jahanbakhshi et al., 2025). Subgroup analyses indicated that VR/exergame interventions demonstrated a numerically larger effect size than traditional exercise modalities in improving cognitive speed (SMD = 1.01 vs. SMD = 0.64). However, this difference should be interpreted cautiously because subgroup analyses reflect effect size differences rather than definitive evidence of superiority. The potential advantage of VR/exergame interventions may be related to their interactive characteristics, which integrate physical activity with visual feedback, rapid response requirements, and continuous engagement with dynamic stimuli. These features may partially overlap with the perceptual and decision-making demands encountered during esports competition, thereby providing additional cognitive stimulation during exercise. Nevertheless, direct comparative trials are still required to determine whether VR/exergame interventions provide greater cognitive benefits than conventional exercise approaches.

The response patterns of players at different competitive levels to exercise interventions may differ fundamentally. Among amateur players, benefits may derive primarily from the interruption of prolonged sedentary states through exercise, alleviating cognitive fatigue via elevation of baseline cortical arousal (De las Heras et al., 2020; Diamond, 2015). Among professional players, benefits may be more prominently reflected in decision-making stability and visual working memory maintenance under conditions of high cognitive load (Lachowicz et al., 2024a; Craig, 2013), representing the marginal contribution of exercise intervention at already high baseline performance levels (Wu et al., 2024). The high heterogeneity observed in the executive function and memory analysis (I^2^ = 86%) was attributed through sensitivity analysis to Lachowicz et al. (2024a), a study with an exceptionally high effect size (SMD = 2.53) and evident deficiencies in experimental control. Following its exclusion, heterogeneity was eliminated and the pooled effect remained significant (SMD = 0.63), further consolidating the reliability of exercise intervention effects at the statistical level.

At the level of neurobiological mechanisms, the findings of this study can be interpreted across three layers. At the molecular level, exercise may promote hippocampal synaptic plasticity remodeling through upregulation of BDNF expression, consistent with the concurrent improvements observed in operational accuracy (SMD = 0.58) and memory function (Yanagisawa et al., 2010). At the systems level, acute exercise can rapidly activate the ascending reticular activating system and enhance prefrontal cortex blood flow perfusion and oxygenation, providing the physiological basis for the significant reduction in reaction time (Erickson et al., 2011). The relatively larger effect size observed for VR/exergame interventions may partly relate to their interactive task characteristics. By combining physical activity with visual feedback, rapid response requirements, and dynamic stimulus processing, these interventions may increase cognitive-motor engagement during exercise. However, because the included studies did not directly evaluate neurophysiological mechanisms, these interpretations remain hypothetical and require confirmation through future studies incorporating objective neural measurements (Craig, 2013).

### 4.3 Limitations

The present study has several limitations. First, although restricting inclusion to randomized controlled trials (RCTs) strengthened the causal inference regarding the effects of exercise interventions and minimized potential selection bias and confounding, this criterion may also limit the generalizability of the findings. Existing RCTs generally involved small sample sizes, and participants were predominantly male and concentrated in specific game genres (mainly FPS and MOBA). Furthermore, the included studies primarily focused on experienced esports players, which may limit the applicability of findings to novice or recreational gamers. Future multicenter studies with larger and more diverse samples are needed to verify the applicability of these findings across different esports populations.

Second, heterogeneity in intervention protocols limits the specificity of exercise recommendations. The included studies varied considerably in exercise modality, intensity, frequency, and duration, and the optimal exercise prescription and dose–response relationship for improving cognitive function in esports players remain unclear. Third, the ecological validity of cognitive assessments remains a concern. Standardized laboratory-based cognitive tests may not fully capture the complex demands of real esports competition, where performance is influenced by psychological state, team interaction, and environmental factors. Future studies should develop assessment approaches that better balance experimental control and ecological validity.

Fourth, evidence regarding long-term effects, underlying mechanisms, and literature retrieval remains insufficient. Most included studies focused on short-term outcomes, and the persistence of cognitive benefits after exercise cessation remains unclear. Moreover, mechanistic interpretations are mainly based on behavioral outcomes without direct neurobiological measurements. Although multiple major databases were systematically searched, Google Scholar was not included, which may have resulted in the omission of some potentially relevant studies. Future research should incorporate longer follow-up periods, broader search strategies, and objective neurobiological assessments to further clarify the long-term effects and mechanisms of exercise interventions in esports players.

### 4.4 Practical implications

The findings of this study provide preliminary evidence-based support for integrating physical activity into esports training systems. Adjunctive exercise interventions may serve as a structural complement to specialized practice by potentially improving players’ cognitive function rather than competing with gaming-specific training. Scheduling moderate acute exercise before training sessions may represent a feasible strategy to optimize reaction speed and decision-making performance during subsequent training, thereby improving the overall efficiency of individual training sessions.

The potential value of VR/exergame interventions for improving cognitive speed warrants further consideration. Given the generally low willingness of esports players to engage in traditional aerobic training, VR-based exercise may represent an engaging alternative that integrates physical activity with interactive cognitive demands. The immersive and visually interactive characteristics of these approaches may increase training engagement and cognitive-motor involvement during exercise. However, whether VR/exergame interventions provide additional cognitive benefits beyond conventional exercise modalities remains to be determined through future direct comparative trials.

From the perspective of long-term career sustainability, regular physical activity over extended periods may help attenuate cognitive fatigue associated with high-intensity training and support players’ overall health. Incorporating physical fitness and neurocognitive indicators into comprehensive player evaluation systems may provide additional reference dimensions for the scientific development of esports talent cultivation and training strategies, contributing to the transition of the industry from experience-driven to evidence-informed approaches.

## 5 Conclusion

Through the systematic integration of existing randomized controlled trial evidence, this study indicates that structured exercise interventions have the potential to positively influence multidimensional cognitive function in esports players. Across the three dimensions of cognitive processing speed, operational accuracy, and executive function and memory, exercise interventions demonstrated consistent beneficial effects, with the improvement in operational accuracy showing high robustness across different intervention modalities and durations. The response patterns to exercise interventions may vary among esports players with different competitive backgrounds, although further research is required to determine whether meaningful differences exist across skill levels. VR/exergame interventions demonstrated numerically larger effects on cognitive processing speed compared with traditional exercise modalities; however, this finding should be interpreted cautiously because the between-subgroup difference was not statistically significant and requires confirmation through future direct comparative studies. Although the current evidence remains limited by sample size, intervention heterogeneity, and study design characteristics, this systematic review provides preliminary support for incorporating structured physical activity into esports training systems. Future research should focus on establishing standardized exercise prescriptions, clarifying dose–response relationships, and directly investigating the neurobiological mechanisms underlying exercise-related cognitive adaptations in esports populations.

## Data Availability

The datasets supporting the conclusions of this meta-analysis are included in the article and its Supplementary files. Search strategies and data extraction forms are available from the corresponding author upon reasonable request.
