# Supplementary material for: Exercise interventions and multidimensional cognitive function in esports players: a systematic review and meta-analysis of randomized controlled trials
Source: Front Psychol. 2026 Aug 14;17:1910084. doi: 10.3389/fpsyg.2026.1910084 (PMC13521916; doi:10.3389/fpsyg.2026.1910084)
Supplement: Supplementary file 1 [file Data_Sheet_1.docx]

Supplementary Figure 1 Subgroup analysis of cognitive speed based on intervention media

Supplementary Figure 2 Subgroup analysis of accuracy based on measurement dimensions

Supplementary Figure 3 Subgroup analysis of accuracy based on intervention types

Supplementary Figure 4 Subgroup analysis of accuracy based on intervention duration

Supplementary Figure 5 Subgroup analysis of executive function and memory based on cognitive domains
