# Supplementary material for: Exercise interventions and multidimensional cognitive function in esports players: a systematic review and meta-analysis of randomized controlled trials
Source: Front Psychol. 2026 Aug 14;17:1910084. doi: 10.3389/fpsyg.2026.1910084 (PMC13521916; doi:10.3389/fpsyg.2026.1910084)
Supplement: Supplementary file 2 [file Data_Sheet_2.docx]

### **Supplementary Text 1 Search Strategies**

**Database: PubMed (via NCBI)**

**Date of Search: May 3, 2026**

| Step | Detailed Refined Search Terms | Results |
| --- | --- | --- |
| #1 | "Video Games"[MeSH Terms] | 184,215 |
| #2 | ("esport*"[tiab] OR "electronic sport*"[tiab] OR "video game*"[tiab]) | 94,831 |
| #3 | #1 OR #2 | 231,450 |
| #4 | "Exercise"[MeSH Terms] | 248,319 |
| #5 | ("exercise"[tiab] OR "physical activity"[tiab] OR "training"[tiab] OR "aerobic"[tiab] OR "resistance"[tiab] OR "HIIT"[tiab] OR "virtual reality"[tiab] OR "exergame*"[tiab]) | 1,142,860 |
| #6 | #4 OR #5 | 1,215,940 |
| #7 | ("randomized controlled trial"[Publication Type] OR "Randomized Controlled Trial"[MeSH Terms]) | 743,150 |
| #8 | ("random*"[tiab] OR "trial"[tiab] OR "group*"[tiab] OR "controlled"[tiab]) | 2,154,620 |
| #9 | #7 OR #8 | 2,410,380 |
| #10 | #3 AND #6 AND #9 | 750 |

#### **Database: Embase (via Elsevier)**

**Date of Search: May 3, 2026**

| Step | Detailed Search Terms | Results |
| --- | --- | --- |
| #1 | 'video game'/exp | 214,560 |
| #2 | ('esport*':ti,ab OR 'electronic sport*':ti,ab OR 'video game*':ti,ab) | 112,480 |
| #3 | #1 OR #2 | 268,140 |
| #4 | 'exercise'/exp | 412,530 |
| #5 | ('exercise':ti,ab OR 'physical activity':ti,ab OR 'training':ti,ab OR 'aerobic':ti,ab OR 'resistance':ti,ab OR 'HIIT':ti,ab OR 'virtual reality':ti,ab OR 'exergame*':ti,ab) | 1,589,420 |
| #6 | #4 OR #5 | 1,723,650 |
| #7 | 'randomized controlled trial'/exp OR 'randomization'/exp | 1,124,590 |
| #8 | ('random*':ti,ab OR 'trial':ti,ab OR 'group*':ti,ab OR 'controlled':ti,ab) | 2,845,130 |
| #9 | #7 OR #8 | 3,124,960 |
| #10 | #3 AND #6 AND #9 | 2,814 |

#### **Database: Web of Science Core Collection (via Clarivate)**

**Date of Search: May 3, 2026**

| Step | Detailed Search Terms | Results |
| --- | --- | --- |
| #1 | TI=(esport* OR "electronic sport*" OR "video game*") | 84,150 |
| #2 | AB=(esport* OR "electronic sport*" OR "video game*") OR AK=(esport* OR "electronic sport*" OR "video game*") | 91,240 |
| #3 | #1 OR #2 | 124,560 |
| #4 | TI=(exercise OR "physical activity" OR training OR aerobic OR resistance OR HIIT OR "virtual reality" OR exergame*) | 412,830 |
| #5 | AB=(exercise OR "physical activity" OR training OR aerobic OR resistance OR HIIT OR "virtual reality" OR exergame*) | 948,150 |
| #6 | #4 OR #5 | 1,084,620 |
| #7 | TI=(random* OR "controlled trial" OR "control group") | 342,150 |
| #8 | AB=(random* OR "controlled trial" OR "control group") | 1,245,390 |
| #9 | #7 OR #8 | 1,384,210 |
| #10 | #3 AND #6 AND #9 | 349 |

#### **Database: The Cochrane Library (via Wiley)**

**Date of Search: May 3, 2026**

| Step | Detailed Search Terms | Results |
| --- | --- | --- |
| #1 | [mh "Video Games"] | 4,320 |
| #2 | (esport*:ti,ab,kw OR "electronic sport*":ti,ab,kw OR "video game*":ti,ab,kw) | 12,840 |
| #3 | #1 OR #2 | 14,150 |
| #4 | [mh "Exercise"] | 24,310 |
| #5 | (exercise:ti,ab,kw OR "physical activity":ti,ab,kw OR training:ti,ab,kw OR aerobic:ti,ab,kw OR resistance:ti,ab,kw OR HIIT:ti,ab,kw OR "virtual reality":ti,ab,kw OR exergame*:ti,ab,kw) | 94,620 |
| #6 | #4 OR #5 | 104,180 |
| #7 | [mh "Randomized Controlled Trial"] | 91,240 |
| #8 | (random*:ti,ab,kw OR trial:ti,ab,kw OR group*:ti,ab,kw OR controlled:ti,ab,kw) | 184,360 |
| #9 | #7 OR #8 | 210,450 |
| #10 | #3 AND #6 AND #9 | 912 |

#### **Database: Scopus (via Elsevier)**

**Date of Search: May 3, 2026**

| Step | Detailed Search Terms | Results |
| --- | --- | --- |
| #1 | ( TITLE-ABS-KEY ( "Video Game" OR "Video Games" OR esport* OR "electronic sport" OR "electronic sports" ) ) | 194,521 |
| #2 | ( TITLE-ABS-KEY ( Exercise OR "physical activity" OR training OR aerobic OR resistance OR HIIT OR "virtual reality" OR exergame* ) ) | 1,412,357 |
| #3 | ( TITLE-ABS-KEY ( "randomized controlled trial" OR random* OR trial OR group* OR controlled ) ) | 2,584,112 |
| #4 | #1 AND #2 AND #3 | 473 |
